# Reply to: clarifications about the article “knowledge, attitudes, and perception of the role of the media regarding COVID-19 in medical students from a peruvian university”

**DOI:** 10.17843/rpmesp.2022.392.11741

**Published:** 2022-06-30

**Authors:** José Luis Paredes, Rafaella Navarro, Jorge Luis Andrade-Piedra, Noemí Hinostroza, Juan Echevarría, Camille Webb

**Affiliations:** 1 Instituto de Medicina Tropical Alexander von Humboldt, Universidad Peruana Cayetano Heredia, Lima, Peru. Universidad Peruana Cayetano Heredia Instituto de Medicina Tropical Alexander von Humboldt Universidad Peruana Cayetano Heredia Lima Peru; 2 Facultad de Medicina, Universidad Peruana Cayetano Heredia, Lima, Peru. Universidad Peruana Cayetano Heredia Facultad de Medicina Universidad Peruana Cayetano Heredia Lima Peru

To the editor. We have received the letter titled: Precisions on the article “Knowledge, attitudes and perception about the role of the media regarding COVID-19 in medical students of a Peruvian university”. We thank the authors for their comments on our study, which we proceed to explain and comment on.

First of all, it is true that there was an error in the wording. The statement in the abstract and in the results of the article should have read: “32% did not know that, in the first five days of illness, serological tests are not preferable for diagnosing COVID-19 compared to molecular tests”. We thank the authors for their comments and have requested correction of this error.

Regarding the second point, the authors mentioned that we must be careful with the concept of herd immunity with respect to COVID-19 in Peru. Herd immunity is calculated using the following formula: 1 / R0, with R0 being the basic reproduction number (R0). This number is the average number of secondary infections caused by a single infectious individual introduced into a fully susceptible population ^1^. Initially, based on data from a study in Wuhan, China, the R0 of SARS-CoV-2 infection was calculated to be 2.2 (95% confidence interval: 1.4 - 3.9) ^2^, which estimated that herd immunity could be reached with 45% of the population immune. As the authors emphasized, knowledge regarding immunity to SARS-CoV-2 infection is constantly evolving. Compared to the first known variant of SARS-CoV-2 (called alpha variant), new variants, such as delta and omicron, have been shown to have higher transmissibility and lower response to vaccine-acquired immunity and prior infection ^3^.

This, as described by the authors, limits the applicability of the concept of herd immunity. This study was conducted during 2021; in this period the knowledge about SARS-CoV-2 infection and about immunity against the different variants of this virus was very limited. In spite of this, in the study we consider that the correct answer is that herd immunity has not been achieved in Peru.
