# Supplementary material for: Reply to: clarifications about the article “knowledge, attitudes, and perception of the role of the media regarding COVID-19 in medical students from a peruvian university”
Source: Rev Peru Med Exp Salud Publica. 2022 Jun 30;39(2):251. doi: 10.17843/rpmesp.2022.392.11741 (PMC11404673; doi:10.17843/rpmesp.2022.392.11741)
Supplement: Translation

# Translation: Réplica a: precisiones sobre el artículo “Conocimientos, actitudes y percepción sobre el rol de los medios de comunicación respecto a la COVID-19 en estudiantes de medicina de una universidad peruana”

**Authors:** José Luis Paredes, Rafaella Navarro, Jorge Luis Andrade-Piedra, Noemí Hinostroza, Juan Echevarría, Camille Webb

**Affiliations:** 1 Instituto de Medicina Tropical Alexander von Humboldt, Universidad Peruana Cayetano Heredia, Lima, Perú.; 2 Facultad de Medicina, Universidad Peruana Cayetano Heredia, Lima, Perú.

Sr. Editor. Hemos recibido la carta titulada: Precisiones sobre el articulo «Conocimientos, actitudes y percepción sobre el rol de los medios de comunicación respecto a la COVID-19 en estudiantes de Medicina de una universidad peruana». Agradecemos a los autores por las observaciones a nuestro estudio, las cuales procedemos a explicar y comentar. 

En primer lugar, es cierto que hubo un error de redacción. El enunciado en el resumen y en los resultados del articulo debieron decir: «el 32% no sabía que, en los primeros cinco días de la enfermedad, las pruebas serológicas no son preferibles para diagnosticar la COVID-19, comparadas con las pruebas moleculares». Agradecemos las observaciones de los autores y hemos pedido la corrección de este error. 

Con respecto al segundo punto, los autores mencionan que debemos ser cuidadosos con el concepto de la inmunidad de rebaño respecto a la COVID-19 en Perú. La inmunidad de rebaño se calcula utilizando la siguiente formula: 1 / R0, siendo R0 el número de reproducción básica (R0). Este número es el promedio de infecciones secundarias causadas por un único individuo infeccioso introducido en una población completamente susceptible ^1^. Inicialmente, a partir de los datos de un estudio en Wuhan, China, se calculó que el R0 de la infección por SARS-CoV-2 era 2,2 (intervalo de confianza al 95%: 1,4 - 3,9) ^2^, con lo que se estimaba que se podría llegar a una inmunidad de rebaño con el 45% de la población inmune. Como los autores recalcaron, el conocimiento respecto a la inmunidad frente a la infección por SARS-CoV-2 se encuentra en una evolución constante. Comparadas a la primera variante conocida del SARS-CoV-2 (llamada variante alfa), nuevas variantes, como la delta y ómicron, han demostrado tener una mayor transmisibilidad y menor respuesta a la inmunidad adquirida por vacunas y por infección previa ^3^.

Esto, como lo describieron los autores, limita la aplicabilidad del concepto de inmunidad de rebaño. Este estudio se realizó durante el 2021; en este periodo el conocimiento sobre la infección por SARS-CoV-2 y sobre la inmunidad frente a las diferentes variantes de este virus era muy limitado. A pesar de esto, en el estudio nosotros consideramos como respuesta correcta el no haber llegado a la inmunidad de rebaño en el Perú.
